# Transplacental Infections Associated with *Macavirus* in Aborted Bovine Fetuses

**DOI:** 10.3390/microorganisms12081608

**Published:** 2024-08-07

**Authors:** Flávia Helena Pereira Silva, Juliana Torres Tomazi Fritzen, Julia Raisa Ximenes Figueiredo, Rafaela Maria Boson Jurkevicz, Ana Flávia Ferrreira Domingues, Milena Patzer Rose, Luara Evangelista Silva, João Luis Garcia, Amauri Alcindo Alfieri, Selwyn Arlington Headley

**Affiliations:** 1 Programa de Pós-Graduação em Biociência Animal, Universidade de Cuiabá, Cuiabá 78060-900, Brazil; 2 Laboratory of Animal Virology, Department of Preventive Veterinary Medicine, Universidade Estadual de Londrina, Londrina 86057-970, Brazil; jufritzen@uel.br (J.T.T.F.);; 3 Laboratory of Parasitology, Department of Preventive Veterinary Medicine, Universidade Estadual de Londrina, Londrina 86057-970, Brazil; rafaela.jurkevicz@uel.br (R.M.B.J.);; 4 Laboratory of Animal Pathology, Department of Preventive Veterinary Medicine, Universidade Estadual de Londrina, Londrina 86057-970, Brazil; 5 Multi-User Animal Health Laboratory (LAMSA), Department of Preventive Veterinary Medicine, Universidade Estadual de Londrina, Londrina 86057-970, Brazil

**Keywords:** diagnostic immunohistochemistry, fetal pathology, *Macavirus*, malignant catarrhal fever, vertical dissemination

## Abstract

The *Macavirus* genus, *Gammaherpesvirinae* subfamily, *Herpesviridae* family, contains ovine gammaherpesvirus 2 (OvGHV2), the cause of sheep-associated malignant catarrhal fever (SA-MCF). Members of the *Macavirus* genus associated with the development of malignant catarrhal fever (MCF) in their respective hosts share the 15A antigenic epitope, are conserved within the DNA polymerase gene and are collectively referred to as the malignant catarrhal fever virus (MCFV) complex. The ability of MCFV and/or OvGHV2 to produce abortions in ruminants is currently unknown, with little documentation of infections by these agents in bovine fetuses. This report presents the findings observed due to the detection of OvGHV2 DNA and MCFV tissue antigens in aborted bovine fetuses from southern Brazil. Four aborted bovine fetuses from three farms, located in a geographical region of Paraná State with elevated immunohistochemical (IHC) prevalence of MCFV tissue antigens, with gestational ages varying between 78 to 208 days were investigated. Significant gross and histopathological alterations were not observed in any of these fetuses. An IHC assay using the 15A-monoclonal antibody (15A-MAb), which is based on the 15A antigenic epitope of *Macavirus*, identified MCFV tissue antigens in multiple organs from two fetuses (#1 and #4); however, positive immunoreactivity to the 15A-MAb IHC assay was not detected in Fetus #2 and #3. Molecular testing amplified OvGHV2 DNA only from the myocardium and lungs of Fetus #1 that had positive intracytoplasmic immunoreactivity to the 15A-MAb IHC assay in these tissues. Furthermore, infections by *Leptospira* spp. were confirmed by molecular assays in fetuses #1, #3, and #4, while PCR detected *Neospora caninum* in the myocardium of Fetus #2. Additionally, molecular assays to identify well-known fetopathy agents of cattle, including bovine viral diarrhea virus, bovine alphaherpesvirus 1, *Histophilus somni*, and *Listeria monocytogenes*, did not amplify the nucleic acids of these pathogens. PCR assays to identify bovine gammaherpesvirus 6 (BoGHV6), another *Macavirus* known to infect cattle in Brazil, were unsuccessful. These findings confirmed that the 15A-MAb IHC assay can be efficiently used to detect MCFV antigens in organs of aborted bovine fetuses. The identification of MCFV antigens with the simultaneous detection of OvGHV2 DNA confirmed that Fetus #1 was infected by OvGHV2 and added to the few descriptions of this infection in aborted fetuses of ruminants worldwide. Moreover, the IHC detection of MCFV in multiple organs of Fetus #4, without the molecular detection of OvGHV2 or BoGHV6, may suggest that this fetus was infected by a *Macavirus* that was not previously diagnosed in cattle herds from Brazil. These findings strongly suggest that OvGHV2 and MCFV can produce transplacental infections in cattle.

## 1. Introduction

The *Macavirus* genus, subfamily *Gammaherpesvirinae*, family *Herpesviridae* [1], contains several members that are associated with the development of disease syndromes in mammalian populations. Members of the *Macavirus* genus include ovine gammaherpesvirus 2 (OvGHV2), alcelaphine gammaherpesvirus 1 and 2 (AlGHV1 and -2), and caprine gammaherpesvirus 2 (CpGHV2) [2,3]. These viruses share the 15A epitope [4], have similarities within the DNA polymerase gene [5], and are collectively referred to as the malignant catarrhal fever virus (MCFV) complex [5,6] since they produce malignant catarrhal fever (MCF) in their respective mammalian hosts [2,3,7]. Furthermore, the 15A antigenic epitope is located within the glycoprotein B gene of *Macavirus* [8]. 

OvGHV2 and AlGHV1 are MCFVs of economic importance worldwide [2,3]; OvGHV2 produces sheep-associated MCF (SA-MCF), while AlGHV1 is related to wildebeest-associated MCF (WA-MCF), with sheep and wildebeest, respectively, acting as asymptomatic reservoir hosts [3,7,9]. Additionally, bovine gammaherpesvirus 6 (BoGHV6) is a *Macavirus* [1] that has not been associated with the development of MCF, and its capability to induce disease in ruminants is considered controversial [10]. Although epidemiological data suggest that OvGHV2 is the only *Macavirus* associated with MCF in ruminants from Brazil [7], the results of recent investigations may indicate the circulation of an undiagnosed *Macavirus* in mammalians from this continental nation [11,12,13]. Thus far, there are no indications of the occurrence of WA-MCF in Brazil.

Although ruminants are infected by OvGHV2 predominantly due to contact with aerosols from asymptomatic sheep, resulting in horizontal transmission [2,14], there are few reports of the identification of OvGHV2 and/or other MCFV in fetal tissues of ruminants [15,16,17], suggesting that MCF can also occur by vertical transmission. Vertical transmission of OvGHV2 was demonstrated in a bovine fetus due to the simultaneous detection of OvGHV2 in fetal and maternal tissues with histological evidence of disease [16]. Additionally, vertical transmission was suspected due to the identification of MCFV antibodies in lambs congenitally infected [15] and the detection of OvGHV2 DNA from the fetuses of cattle [18,19] and buffalo [17]. Furthermore, vertical transmission of AlGHV1 was experimentally demonstrated in wildebeest [20], while cows [21] and 50% (47/94) of placental tissues of wildebeest contained low viral DNA loads of AlGHV1 [22]. These findings suggest that fetal and placental material may be associated with the transmission of MCFV from infected wildebeest to susceptible cattle populations [23]. Additionally, BoGHV6 was identified in fetal tissues of cattle simultaneously infected with *Histophilus somni* [10].

Collectively, these findings raised the question of the epidemiological importance of vertical transmission of *Macavirus* and its possible role in the dissemination of infections to susceptible mammalian hosts. Accordingly, the objectives of this study were to identify *Macavirus* in fetal tissues of cattle from southern Brazil and provide additional demonstration of vertical infection by this complex.

## 2. Materials and Methods

### 2.1. Study Location, Gross Evaluation, and Collection of Fetal Material

Between August 2022 and July 2023, four bovine fetuses from three farms were received at the Laboratory of Animal Pathology, Universidade Estadual de Londrina, for pathological diagnosis. All farms were in the rural region of Londrina, Paraná, southern Brazil, and there were reports of repeated bovine abortions at these establishments. Cattle at these farms were routinely immunized against common bovine reproductive disease agents, including bovine viral diarrhea virus (BVDV), bovine alphaherpesvirus 1 (BoAHV1), *Brucella abortus*, and *Leptospira* spp., with inactivated vaccines. Furthermore, these farms are within the mesoregion of Paraná State where 41% (23/56) of cattle with renal lesions contained MCFV antigens [24]. Additionally, the owner of farm #1 related that sheep were not reared on his property but were reared close to his farm; also, sheep were not reared on farms #2 and #3 or within neighbouring establishments.

The average gestational period of each fetus was estimated by measuring the distance between the crown and rump as described [25,26]. All fetuses were submitted to routine *post-mortem* evaluations soon after arrival. Tissue sections of the lungs, kidneys, myocardium, spleen, brain, liver, small intestine, and thymus of all fetuses were collected for routine histopathological evaluation with the hematoxylin and eosin stain. Selected formalin-fixed paraffin-embedded (FFPE) sections of these organs were used for immunohistochemistry (IHC). Duplicate sections of these organs were maintained at −80 °C until used in molecular assays.

### 2.2. Immunohistochemical Detection of Malignant Catarrhal Fever Virus Antigens

The FFPE tissue sections were used in IHC assays designed to detect intralesional tissue antigens of MCFV with the 15A-monoclonal antibody (15A-MAb) using the previously described protocol [27]. This IHC assay is based on the detection of the 15A epitope of members of the *Macavirus* genus known to cause MCF [4,5]. Positive controls consisted of FFPE tissue sections derived from ruminants known to contain intralesional antigens of OvGHV2 [27]. The negative controls were achieved by substituting the 15A-MAb with its diluent and the application of the 15A-MAb on FFPE tissue sections known not to contain tissue antigens of MCFV. Positive and negative controls were used in each IHC assay. 

### 2.3. Molecular Investigation of Pathogens Associated with Reproductive Disease of Cattle

Nucleic acids from selected tissue fragments (liver, spleen, myocardium, small intestine, thymus, and lungs) of each fetus collected fresh during *post-mortem* were extracted using a combination of the phenol/chloroform/isoamyl alcohol and silica/guanidine isothiocyanate methods as described [28,29]. These suspensions were then used in molecular assays designed to detect nucleic acids of the common infectious agents of bovine reproductive disease, using the previously described protocols for BoAHV1, BVDV, *Histophilus somni*, *Leptospira* spp., *Listeria monocytogenes*, and *Neospora caninum*. Furthermore, molecular assays were also performed to detect OvGHV2 and BoGHV6 since both *Macavirus* were previously detected in fetal tissues of cattle [10,16]. Additionally, the degenerate pan-herpesvirus PCR assay for the detection of a wide range of herpesvirus was also performed [30]. A list of the target genes, primers, and amplicon size of the molecular assays used during this study to identify infectious disease pathogens associated with reproductive diseases of ruminants is provided (Table 1).

Positive controls consisted of DNA/RNA from previous cases [9,26,30]. Nuclease-free water (Invitrogen, Carlsbad, CA, USA) was used as the negative control in all PCR assays; positive and negative controls were included in all molecular assays. All PCR products were separated by electrophoresis in 2% agarose gels, stained with ethidium bromide, and examined under ultraviolet light.

### 2.4. Sequencing of the OvGHV2 Tegument Protein Gene and Phylogenetic Evaluations

The products derived from the OvGHV2 heminested PCR assays were purified using the PureLink^®^ Quick Gel Extraction and PCR Purification Combo Kit (Invitrogen^®^ Life Technologies, Carlsbad, CA, USA), quantified by using a Qubit^®^ Fluorometer (Invitrogen^®^ Life Technologies, Eugene, OR, USA), and submitted to direct sequencing in both directions with the forward and reverse primers used in the respective molecular assays in an ABI3500 Genetic Analyzer sequencer with the BigDye Terminator v3.1 Cycle Sequencing Kit (Applied Biosystems^®^, Foster City, CA, USA). 

Sequence quality analyses and consensus sequences were obtained using PHRED and CAP3 homepage (http://asparagin.cenargen.embrapa.br/phph/ (accessed on 21 September 2023), respectively. Similarity searches of the OvGHV2 tegument protein gene were performed with nucleotide (nt) sequences deposited in GenBank using the basic local alignment search tool (https://blast.ncbi.nlm.nih.gov/Blast.cgi (accessed on 21 September 2023).

The nt sequences derived from this study were compared with the reference strain of OvGHV2 and strains of OvGHV2 identified in ruminants from Brazil, as well as the reference strains for AlGHV1 and 2. The nt sequence identity matrices were constructed using the BioEdit software version 7.0.8.0. The phylogenetic tree was reconstructed using the Neighbor-joining method with the Kimura 2-parameter model, based on 1000 bootstrapped datasets.

## 3. Results

### 3.1. Biological Data and Pathological and Immunohistochemical Findings

Three of the fetuses (#1, 2, and 4) evaluated were from the dairy breed of cattle; one fetus was in the first gestational trimester (#2) and three (#1, 3, and 4) were within the third trimester (Table 2). Significant gross alterations were not observed in any of the fetuses, with moderate periglomerular hemorrhage being the only histopathological finding diagnosed in Fetus #1. 

**Figure 1:**
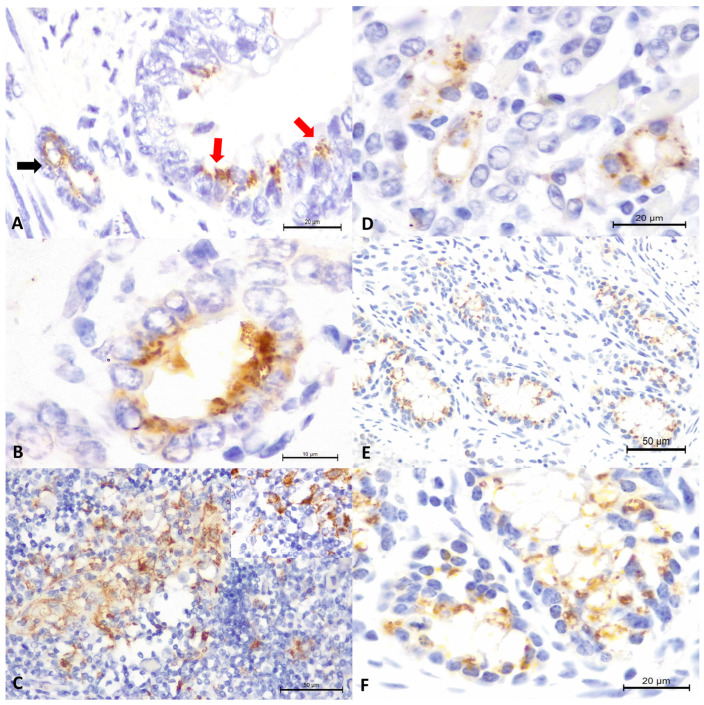
Immunohistochemical detection of malignant catarrhal fever virus antigens in aborted bovine fetuses. There is positive intracytoplasmic immunoreactivity within epithelial cells of the bronchus (black arrow) and bronchial gland (red arrows) of the lungs (**A**), within the bile duct epithelium of the liver (**B**), and within thymic lymphocytes; closer view at the insert (**C**), renal tubules (**D**), and cryptal epithelium cells of the small intestine (**E**,**F**). Immunoperoxidase counterstained with hematoxylin. Bars, (**A**,**D**,**F**), 20 µm; (**B**), 10 µm; (**C**,**E**), 50 µm; insert, 100× Obj.

There was positive intracytoplasmic immunoreactivity to the 15A-MAb IHC in several tissues of Fetus #1 and #4, while MCFV antigens were not identified in any tissues from Fetus #2 and 3 (Table 1). The positive immunoreactivity to the 15A-MAb was similar in these two fetuses (#1 and 4), with immunoreactivity being identified within bronchial epithelial cells and peribronchial glands of the lungs (Figure 1A), bile duct epithelium (Figure 1B), and lymphocytes of the thymus (Figure 1C) of these fetuses. Additionally, there was positive intracytoplasmic immunoreactivity to the 15A-MAb within epithelial cells of the renal tubules in Fetus #1 (Figure 1D) that had periglomerular hemorrhage by histopathology and within epithelial cells of the intestinal crypts of Fetus #4 (Figure 1E,F).

### 3.2. Molecular Characterization and Sequencing of OvGHV2 and Phylogenetic Analyses

The heminested Baxter PCR [31] amplified the desired amplicon from the lungs with positive immunoreactivity to the 15A-MAb IHC assay and the myocardium of Fetus #1; direct sequencing confirmed these results. The strain of OvGHV2 derived from this study is named PR/UEL-496 and is deposited in GenBank (Accession #OR761839). 

The partial nt sequence of the OvGHV2 tegument protein gene derived from this study has 99% nt sequence identity with the prototype strain of OvGHV2 (NC007646), 100% with the complete genome (DQ198083) and the partial fragment (MZ221210) identified in sheep. Furthermore, a nt homology of 99.5% occurred with strains of OvGHV2 identified in cows (JQ780445; KJ658293) and a bovine fetus (KJ658294) from different geographical regions of Brazil. Also, a 99% nt sequence identity was detected with the strain of OvGHV2 identified in a goat (OK490363) from Brazil. Additionally, the OvGHV2 strain herein identified had 49.3% and 45.2% homology with the prototype strains of AlGHV2 (NC024382) and AlGHV1 (NC002531), respectively.

**Figure 2:**
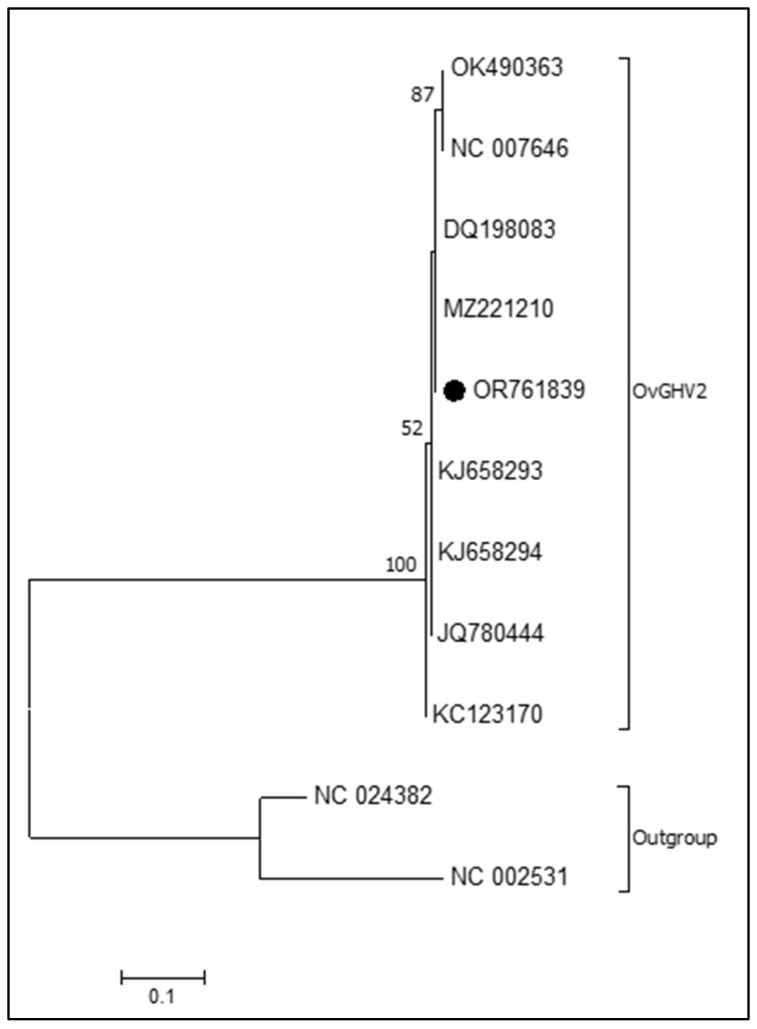
Phylogenetic tree based on 221 base pairs of the OvGHV2 tegument protein. The sequence derived from this study is highlighted (●). The analyses were based on the Neighbor-joining method from the Kimura 2-parameter model; bootstrapping was statistically supported with 1000 replicates using MEGA 7.0. The strains evaluated are identified by their GenBank accession numbers; AlGHV1 and 2 were included as the outgroup.

The phylogenetic tree (Figure 2) revealed that all strains of OvGHV2 formed a distant cluster from AlGHV1- and 2. Furthermore, small clusters were formed with the closely related strains of OvGHV2, with the strain herein identified grouping with the strains identified in sheep from (MZ221210) Brazil and the USA (DQ198083). 

### 3.3. Molecular Identification of Reproductive Pathogens of Ruminants

Nucleic acids of *Leptospira* spp. were detected in fetuses from all farms (Table 1); being identified in the small intestine of Fetus #1, thymus of fetuses #1 and 3#, and the lungs and liver of Fetus #4. Furthermore, *N. caninum* DNA was detected only in the myocardium of Fetus #2. Consequently, dual infections were identified in fetuses #1 (OvGHV2 and *Leptospira* spp.) and #4 (MCFV and *Leptospira* spp.), with singular infections due to *N. caninum* and *Leptospira* spp. diagnosed in fetuses #2 and #3, respectively (Table 1). Additionally, all other reproductive disease pathogens of cattle investigated (BVDV, BoAHV1, *H. somni*, and *L. monocytogenes*), as well as BoGHV6, were not detected by their respective molecular assays. Furthermore, nucleic acids were not detected with the degenerated primers designed to amplify a wide range of herpesvirus.

## 4. Discussion

The 15A-MAb IHC assays identified MCFV tissue antigens in multiple organs of fetuses #1 and #4, confirming that these fetuses were infected by an MCFV. As far as the authors are aware, these findings may represent one of the few studies to demonstrate MCFV tissue antigens in ruminant fetuses and probably the first using IHC; earlier studies identified neutralizing antibodies to the MCFV in wildebeest [20,21]. The identification of MCFV antigens by IHC in several fetal tissues of cattle during this study using the 15A-MAb is an important and exciting finding since it demonstrates that this IHC assay can be effectively used to identify MCFV antigens even in the absence of significant histopathological alterations in the affected tissues and opens the way for the utilization of this IHC assay in retrospective studies based on archival samples of fetal ruminant tissues. 

The phylogenetic analyses demonstrated that the strain herein identified had 99–100% nt sequence homology with the reference strain of OvGHV2 and other strains derived from ruminants in Brazil and the USA, with the formation of a large cluster of OvGHV2 strains. Additionally, the two types of *Macavirus* formed distinct clusters. Similar findings were reported worldwide [5,6,16,39], demonstrating that these viruses are phylogenetically related, with the OvGHV2 strains being well conserved within the tegument protein gene [5,39]. 

During this study, the nucleic acids of several well-known fetopathic agents of ruminant abortion [40,41], including BVDV, BoAHV1, *H. somni*, and *L. monocytogenes* were not detected, suggesting that these were not associated with the abortive effects of the four fetuses on the three farms. However, fetuses #1 and #4 were simultaneously infected by *Leptospira* spp.; concomitant infections were previously described in bovine fetuses infected by BoGHV6 and *H. somni* [10], and in bovine fetuses with pneumonia [42]; these infections may be commonly diagnosed in aborted bovine fetuses [40]. Therefore, simultaneous infections in aborted bovine fetuses may be more frequent than described in the literature. However, fetuses #2 and #3 were infected only by *N. caninum* and *Leptospira* spp., respectively; infections in aborted bovine fetuses seem to be more frequently associated with single infectious agents [40]. Collectively, these findings suggest that abortions on these farms were probably associated with infections by *Leptospira* spp. and *N. caninum*. However, since the MCFV can induce tissue destruction due to dysregulated cytotoxic lymphocytes [2,14], the possible immunosuppressive effects of this complex on the occurrence of these simultaneous infections cannot be ignored.

### 4.1. Ovine Gammaherpesvirus 2 and MCFV Can Produce Infections in Bovine Fetuses 

The amplification of OvGHV2 from several tissues of Fetus #1 suggests that this fetus was infected by this virus; similar findings were previously described [16,17,18,19]. Furthermore, the identification of MCFV antigens in the tissues of Fetus #4 by IHC, without the simultaneous molecular detection of OvGHV2 or BoGHV6, suggests that this fetus could have been infected by another *Macavirus,* considering that the 15A epitope is common to all MCFV associated with the development of MCF [4,5,6]. These findings are in accordance with the recommendations for fetal pathology where infections are associated with the detection of tissue antigens or nucleic acids within fetal tissues [40]. A novel *Macavirus* was recently detected in Roan antelopes due to viral amplification by Sanger sequencing but without the simultaneous detection of viral proteins by in situ hybridization [43]. Therefore, with the utilization of advanced diagnostic techniques, undiagnosed Macaviruses are likely to be identified. 

During this study, Fetus #1 was maintained on a farm where sheep were reared within proximity. In contrast, sheep rearing was not practiced on farm C or within the neighbouring farms—sheep are the asymptomatic hosts for OvGHV2 [7,9]. Additionally, both farms are located within the mesoregion of Paraná State where 41% of cattle with renal lesions were infected by a MCFV [24]. These findings are of extreme epidemiological importance and may suggest that the MCFV infection identified in Fetus #4 from farm C by IHC may be associated with infection not by OvGHV2, but by another *Macavirus* not previously diagnosed in Brazil. Similar findings were recently described in a goat [11] and in cattle simultaneously infected by OvGHV2 and *Macavirus* that were intoxicated due to the ingestion of *Brachiaria* spp. grass [12]. In that study, we postulated that infections confirmed by the IHC detection of MCFV tissue antigens of cattle without the simultaneous amplification of OvGHV2 and BoGHV6 DNA may be associated with the circulation of a previously undiagnosed *Macavirus* in cattle herds from Brazil [12]. Interestingly, a serological study identified OvGHV2 in 39.5% (17/43) of dairy farms from southern Brazil that had no contact with sheep (S.A. Headley, personal communication). These are novel findings since epidemiological data suggest that only OvGHV2 has been associated with the development of MCF in ruminants from Brazil [7]. Collectively, these findings demonstrate that there is consistent evidence to indicate that OvGHV2 and MCFV can produce transplacental transmission in cattle and that there may be an undiagnosed *Macavirus* circulating in cattle herds from Brazil. 

The confirmation of infection by OvGHV2 in Fetus #1 adds to the few cases worldwide that have detected this pathogen in the fetal tissues of ruminants [16,17,18,19]. A previous report from our group identified OvGHV2 in the fetal and maternal tissues of a cow with histopathological evidence of SA-MCF, thereby confirming transplacental transmission [16]. However, during the current study, maternal tissues or blood were not available to confirm infection in the cow. Nonetheless, since OvGHV2 is not a commensal pathogen in cattle [2,3,7], the identification of OvGHV2 DNA in fetal tissues of a cow suggests that infection was vertical, i.e., transferred from the infected cow to her fetus. Collectively, these results demonstrate that OvGHV2 can produce intrauterine infections in fetuses of cattle. Furthermore, experimental data have demonstrated the possibility of transmission of OvGHV2 via the male genital tract due to elevated viral loads detected in the epididymis, prostate, and vesicular gland of sheep [44]. Nevertheless, the epidemiological importance, if any, of these results will only be appreciated after the investigation of a larger number of aborted fetal tissues of cattle to identify additional evidence of fetal infections by OvGHV2 or MCFV.

### 4.2. The Importance of the Crown-Rump Measurement of Bovine Fetuses

Abortion in cattle is characterized by the expulsion of a fetus between the completion of differentiation (day 42) and the limit of fetal viability development (day 260) outside of the uterus [25,26,45]. The estimated gestational age (ranging from 78–205 days) of the fetuses from this study, using the crown-rump measurement, confirmed that the fetuses evaluated during this investigation were aborted. Therefore, it is recommended that in studies that evaluate bovine fetuses, the crown-rump measurement protocol be used to effectively differentiate between aborted fetuses from embryonic death, which occurs at up to around 45 days [25,26]. It must be highlighted that the time established to determine complete fetal differentiation (day 42) coincided with the classical timing of manual pregnancy detection in ruminants when this definition was formulated [45]. However, with the utilization of ultrasonographic diagnosis [46], fetal death can be observed as early as 30 days [45] but is normally detected between 35 and 40 days of gestation in the absence of a fetal heartbeat [46].

### 4.3. Study Limitations and Future Perspectives

A limiting factor during this study was the non-amplification of herpesvirus DNA in Fetus #4 with the degenerate consensus herpesvirus primers [30]. This was because this fetus contained antigens of a *Macavirus* due to the IHC detection with the 15A-MAb assay, but OvGHV2 or BoGHV6 DNA was not detected by their respective molecular assays. The consensus primers for the diagnosis of MCFV were designed to amplify orthologue genes from related organisms, resulting in the characterization of novel pathogen species or strains via phylogenetic analysis [47]. However, consensus primers are not perfect for the amplification of all organisms since they may demonstrate low specificity and sensitivity when used to detect distantly related genes or when there is a low viral load in the sample that is being evaluated [47]. 

Therefore, in this specific case, the non-amplification of herpesvirus DNA with the consensus primers may be due to several factors, including (a) an extremely reduced viral load in the tissues evaluated; (b) the presence of a very distant member of the *Macavirus* genus in the tissue not detectable by the degenerate primers, considering that the 15A-MAb IHC detected tissue antigens of a *Macavirus* in Fetus #4; or (c) the non-amplification by the consensus PCR assay of adequate copies of DNA to be observable in the agarose gel. Accordingly, the classification of the infection in Fetus #4 as MCFV-associated is recommended [13] since neither OvGHV2 nor BoGHV6 DNA was detected in the fetus that contained positive immunoreactivity to the 15A IHC assay in multiple tissues. This is additional evidence to indicate the possible circulation of an undiagnosed *Macavirus* in cattle herds from Brazil. 

A major limiting factor during this study was the small number of fetuses evaluated since a larger number of fetal tissues would have provided more consistent results. However, the main problem working with fetal pathology is obtaining viable fetal tissues for histopathological evaluation since most bovine fetuses received are frequently in some form of *post-mortem* autolysis. This can be related to the routine activities on these farms since cases of aborted fetuses are not frequently identified, collected, and submitted immediately after expulsion for routine diagnostic evaluations. *Post-mortem* autolysis of bovine fetuses may also be related to the time of death within the uterus, principally in cases of intrauterine death [45]. Nonetheless, the results of the limited number of fetuses herein evaluated did not affect the main results, i.e., the demonstration that OvGHV2 and MCFV can induce vertical infections in cattle. 

Since there is emerging evidence to suggest the possible circulation of an undiagnosed *Macavirus* in cattle herds from Brazil, studies are being implemented to determine its possible existence and epidemiological importance in the understanding of MCF in this continental nation. Consequently, the utilization of next-generation sequencing and/or Sanger sequencing to examine the virome of these viruses may be useful in determining the existence of an undiagnosed *Macavirus* in cattle herds from Brazil. It must be highlighted that the identification of MCFV is very challenging, not only in the asymptomatic hosts but also in determining the extent of dead-end hosts [43] that can be infected by these viruses. Additionally, there are several grey areas associated with the epidemiology and dissemination of OvGHV2 as well as the occurrence of SA-MCF in ruminants from Brazil that need to be investigated. 

## 5. Conclusions

OvGHV2 was identified in organs of one aborted bovine fetus that had MCFV antigens in multiple tissues, while MCFV antigens were detected with the 15A-MAb IHC assay in several organs of a fetus without the simultaneous molecular detection of a *Macavirus* of cattle that was previously diagnosed in Brazil. Furthermore, both fetuses infected with *Macavirus* were concomitantly infected by *Leptospira* spp. Although abortions on these farms were most likely due to infections by *N. caninum* and *Leptospira* spp., these findings present additional evidence to demonstrate that OvGHV2 and MCFV can produce transplacental infections in bovine fetuses.

## Figures and Tables

**Table 1:** List of primers with target genes and amplicon size of the molecular assays used to identify infectious disease pathogens associated with reproductive diseases of ruminants.

	Target Genes	Primer Sequences (5′-3′)	Amplicon Size (bp)	References
Ovine gammherpesvirus 2	Tegument protein	Fw-AGTCTGGGTATATGAATCCAGATGGCTCTC	422	[31]
		Rv-AAGATAAGCACCAGTTATGCATCTGATAAA		
Bovine gammaherpesvirus 6	DNA polymerase	Fw-ACAGACGGGCAGCAGATAAG	551	[32]
		Rv-ATGGTTCGCCCCTGTAGAGT		
		Rv-TGTGGGTGCGAGTTCTGC (2nd round)		
Pan herpes	DNA polymerase	Fw-GAYTTYGCNAGYYTNTAYCC Fw-TCCTGGACAAGCAGCARNYSGCNMTNAA Rv-GTCTTGCTCACCAGNTCNACNCCYTT Fw-TGTAACTCGGTGTAYGGNTTYACNGGNGT (2nd round) Rv-CACAGAGTCCGTRTCNCCRTADAT (2nd round)	variable	[30]
Bovine viral diarrhea virus	5’UTR	Fw-ATGCCCWTAGTAGGACTAGCA	288	[33]
		Rv-TCAACTCCATGTGCCATGTAC		
		Rv-GCTAGTTCTGTGGTGGATTGTTGTC (2nd round)		
BoAHV1	Glycoprotein C	Fw-CAACCGAGACGGAAAGCTCC	354	[34]
		Rv-AGTGCACGTACAGCGGCTCG		
*Histophilus somni*	16S	Fw-GAAGGCGATTAGTTTAAGAG	400	[35]
		Rv-TTCGGGCACCAAGTRTTCA		
*Leptospira* spp.	*rrs2*	Fw-GGCGGCGCGTCTTAAACATG	331	[36]
		Rv-TTCCCCCCATTGAGCAAGATT		
*Listeria monocytogenes*	Listeriolysin	Fw-GCATCTGCATTCAATAAAGA	174	[37]
		Rv-TGTCACTGCATCTCCGTGGT		
*Neospora caninum*	NC-5	Fw-CCCAGTGCGTCCAATCCTGTAAC Rv-CTCGCCAGTCAACCTACGTCTTCT	337	[38]

**Table 2:** Immunoreactivity to malignant catarrhal fever virus antigens with molecular detection of *Macavirus* and fetopathic agents in aborted bovine fetuses.

Farms	Bovine Fetuses #	Breed	Estimated Gestational Period (Days)	MCFV IHC Immunoreactivity	*Macavirus* PCR		*Leptospira* spp.	*Neospora* *caninum*	Types of Infection
					OvGHV2	BoGHV6			
A	1	Girolando	189	Liver, lungs, kidney, thymus	Myocardium, lungs	Not amplified	Small intestine, thymus	Not amplified	OvGHV2 + *Leptospira* spp.
	2	Girolando	78	Not detected	Not amplified	Not amplified	Not amplified	Myocardium	*Neospora caninum*
B	3	Nellore	200	Not detected	Not amplified	Not amplified	Thymus	Not amplified	*Leptospira* spp.
C	4	Jersey	205	Liver, lungs, small intestine, thymus	Not amplified	Not amplified	Lung, liver	Not amplified	MCFV + *Leptospira* spp.

Legend: MCFV IHC, malignant catarrhal fever virus immunohistochemistry; OvGHV2, ovine gammaherpesvirus 2; BoGHV6, bovine gammaherpesvirus 6.

## Data Availability

The partial nucleotide sequence of the OvGHV2 strain identified during this study is deposited in GenBank (https://www.ncbi.nlm.nih.gov/genbank/ (accessed on 21 September 2023)). Name of strain: OvGHV2/PR/UEL-496 (GenBank Accession #OR761839).
